# Discovery and *in Vivo* Evaluation of Novel RGD-Modified Lipid-Polymer Hybrid Nanoparticles for Targeted Drug Delivery

**DOI:** 10.3390/ijms151017565

**Published:** 2014-09-29

**Authors:** Yinbo Zhao, Dayong Lin, Fengbo Wu, Li Guo, Gu He, Liang Ouyang, Xiangrong Song, Wei Huang, Xiang Li

**Affiliations:** 1 State Key Laboratory of Biotherapy, Department of Pharmacy and Urology, West China Hospital, Sichuan University, Chengdu 610041, China; E-Mails: sklb_zhaoyinbo@126.com (Y.Z.); wufengbo_@163.com (F.W.); ouyangliang@scu.edu.cn (L.O.); xiangrong_11@126.com (X.S.); 2 Department of Anesthesiology, Sichuan Academy of Medical Sciences & Sichuan Provincial Peopleʼs Hospital, Chengdu 610072, China; E-Mail: dyl_spph@126.com; 3 West China School of Pharmacy, Sichuan University, Chengdu 610041, China; E-Mail: guoli@scu.edu.cn; 4 State Key Laboratory Breeding Base of Systematic Research, Development and Utilization of Chinese Medicine, Chengdu University of Traditional Chinese Medicine, Chengdu 611137, China; E-Mail: huangwei@cdutcm.edu.cn

**Keywords:** curcumin, lipid-shell and polymer-core hybrid nanoparticle (lpNP), PLGA, RGD

## Abstract

In the current study, the lipid-shell and polymer-core hybrid nanoparticles (lpNPs) modified by Arg–Gly–Asp(RGD) peptide, loaded with curcumin (Cur), were developed by emulsification-solvent volatilization method. The RGD-modified hybrid nanoparticles (RGD–lpNPs) could overcome the poor water solubility of Cur to meet the requirement of intravenous administration and tumor active targeting. The obtained optimal RGD-lpNPs, composed of PLGA (poly(lactic-*co*-glycolic acid))–mPEG (methoxyl poly(ethylene- glycol)), RGD–polyethylene glycol (PEG)–cholesterol (Chol) copolymers and lipids, had good entrapment efficiency, submicron size and negatively neutral surface charge. The core-shell structure of RGD–lpNPs was verified by TEM. Cytotoxicity analysis demonstrated that the RGD–lpNPs encapsulated Cur retained potent anti-tumor effects. Flow cytometry analysis revealed the cellular uptake of Cur encapsulated in the RGD–lpNPs was increased for human umbilical vein endothelial cells (HUVEC). Furthermore, Cur loaded RGD–lpNPs were more effective in inhibiting tumor growth in a subcutaneous B16 melanoma tumor model. The results of immunofluorescent and immunohistochemical studies by Cur loaded RGD–lpNPs therapies indicated that more apoptotic cells, fewer microvessels, and fewer proliferation-positive cells were observed. In conclusion, RGD–lpNPs encapsulating Cur were developed with enhanced anti-tumor activity in melanoma, and Cur loaded RGD–lpNPs represent an excellent tumor targeted formulation of Cur which might be an attractive candidate for cancer therapy.

## 1. Introduction

Curcumin (Cur) is a natural polyphenolic phytomedicine known as diferuloylmethane (1,7-bis(4-hydroxy-3-methoxyphenyl)-1,6-heptadiene-3,5-dione) [1]. Cur is the active component of turmeric, which is widely used as spice and traditional medicine in southeast Asian countries [2]. In previous reports, Cur was shown to have anti-inflammatory, anti-oxidant, anti-bacterial, anti-virus, anti-tumor, and hyperlipidemic activities [3,4,5]. However, the therapeutic efficacy of Cur is limited by its poor solubility [6], extensive first pass metabolism, and poor oral bioavailability, and thus calls for a tumor-targeted formulation with controlled release property [7].

Lipid-shell and polymer core nanoparticles (lpNPs) bear the dual advantages of polymer nanoparticles and liposomes. The combination of high biocompatibility, stability, and favorable pharmacokinetic profile make the lpNPs promising drug carriers [8]. The drug can be encapsulated in the polymer core and/or in the lipid bilayers. Thereby, the drug loading efficiency and the load capacity is increased [9]. The current preparation strategies for fusion particles (e.g., lipopolyplexes) contain several conventional synthetic methods, which are difficult to scale up, and result in low efficiency in drug delivery system development [10,11,12,13]. Recently, Chan *et al.* developed a protocol for the self-assembly of nanoparticles (NPs) that combine the properties of liposomes and polymeric NPs [14].

Although the drug loaded in lpNPs accumulated in tumor sites through the enhanced permeability and retention (EPR) effects between the tumor and normal tissues, the introduction of the active targeting strategy for increasing the uptake efficiency of the entrapped anticancer drugs is still necessary [15]. Messerschmidt *et al.* described targeted lpNPs, which are composed of an inner single-chain tumor necrosis factor (scTNF)-functionalized polymeric core, coated by a lipid shell endowed with polyethylene glycol (PEG) chains for steric stabilization and a single-chain antibody fragment (scFv) fragment for targeting. Using the scFv as an active targeted fragment against the tumor stroma marker, the fibroblast activation protein (FAP) and the scTNF-lpNPs could specifically bind to FAP expressing cells and hardly bind to FAP-negative cells [16].

Integrin is a family of cell surface receptors responsible for anchoring cells to the extracellular matrix (ECM). They are over-expressed in melanoma tumor cells and tumor endothelial cells [17]. The Arg–Gly–Asp (RGD) sequence can bind to several integrins such as integrin α_ν_β_3_ and α_v_β_1_ [18]. A linear RGD peptide has been used to construct an amphiphilic polymer cholesterol–poly(ethylene glycol)–RGD peptide (Chol–PEG–RGD). To establish a RGD-modified liposomal drug delivery system of Cur, a natural anti-tumor agent for treating a variety of solid tumor cells was used [19]. In the present manuscript, we employed lpNPs with a steric stabilized PEG–lipid-shell, and a targeting moiety, via the insertion of Chol–PEG–RGD into the lipid coat. We showed that these modifications diminished non-specific adsorption of the particles to the surface of mammalian cells and mediate selective delivery to angiogenesis or integrin receptor positive target cells. Furthermore, lipid coating reduced the *in vitro* off-target cytotoxicity of the lpNPs.

## 2. Results and Discussion

### 2.1. Characterization of Curcumin (Cur)–Lipid-Shell and Polymer Core Nanoparticles (lpNPs)

**Figure 1:**
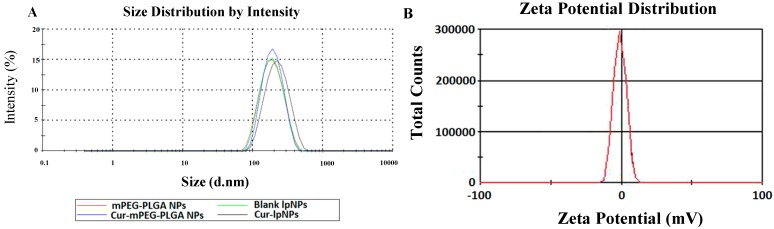
Characterization of Cur–lpNPs. (**A**) particle size of Cur–lpNPs; (**B**) zeta potential of Cur–lpNPs.

Cur–lpNPs were prepared using a simple and controllable double emulsification method described in our previous reports. In the optimized Cur loaded lpNPs, the payload of Cur in the lpNPs was 5% *w*/*w*, and the drug loading (DL) and encapsulation efficiency (EE) were 4.80% ± 0.03% and 96.0% ± 0.6%, respectively (Table 1). Furthermore, the particle size, polydispersity index (PDI), and zeta potential of the obtained Cur–lpNPs were 216.6 ± 4.7 nm, 0.205 ± 0.02, and −0.23 ± 0.12 (mV), respectively (Figure 1A,B).

**Table 1:** Drug loading (DL) and encapsulation efficiency (EE) results of curcumin (Cur) –lipid-shell and polymer core nanoparticles (lpNPs) with Cur loading content between 2% and 7%.

Drug/Polymer (%, *w*/*w*)	Size (nm)	Polydispersity Index (PDI)	DL (%)	EE (%)
2	201.9 ± 7.8	0.195 ± 0.05	2.0 ± 0.01	100 ± 0.24
3	206.36 ± 6.1	0.192 ± 0.07	2.96 ± 0.02	98.67 ± 0.67
4	202.4 ± 5.2	0.202 ± 0.03	3.91 ± 0.02	97.75 ± 0.5
5	216.6 ± 4.7	0.205 ± 0.02	4.80 ± 0.03	96.0 ± 0.6
7	260.5 ± 8.1	0.282 ± 0.07	4.95 ± 0.07	70.71 ± 1.0

**Figure 2:**
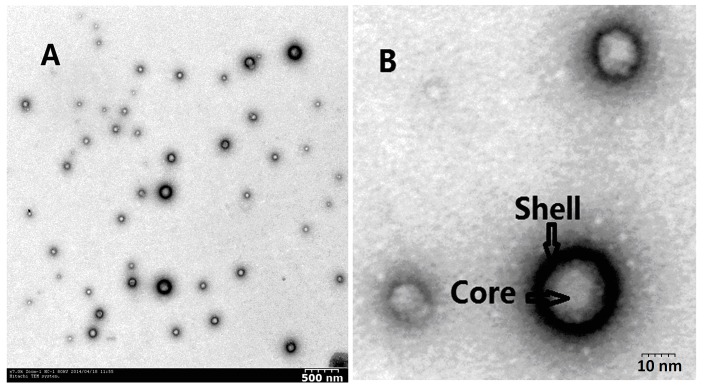
(**A**) Typical TEM images of Cur-lpNPs; (**B**) Expand TEM images of Cur-lpNPs.

The free Cur formed a turbid suspension in the aqueous solution due to its hydrophobicity, whereas a transparent solution of Cur-lpNPs was observed. The TEM image of Cur-lpNPs is exhibited in Figure 2A,B, and it shows that Cur-lpNPs are spherical in shape, with a diameter of about 200 nm. The results of particle size analysis and microstructure of Cur observed by TEM suggest that a homogenous and stable solution of Cur-lpNPs could be obtained by encapsulating Cur into lipid-shell and polymer core nanoparticles.

### 2.2. In Vitro Cytotoxicity Evaluation

**Figure 3:**
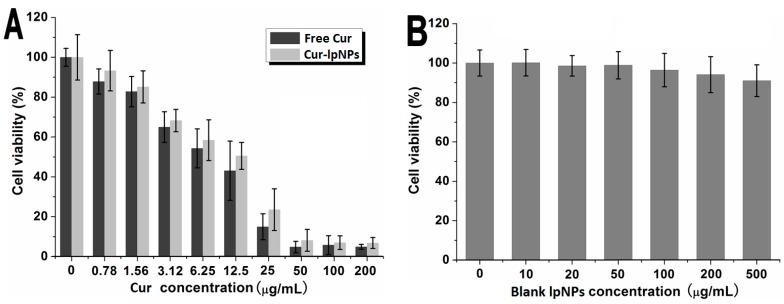
Cytotoxicity of Cur–lpNPs, free Cur and blank lpNPs. (**A**) Cytotoxicity evaluation of Cur–lpNPs and free Cur on B16 cells; (**B**) Cytotoxicity study of blank lpNPs on HEK293 cells.

The *in vitro* cytotoxicity of Cur–lpNPs and free Cur was determined via cell viability assay on B16 cells, and the results are shown in Figure 3A. As the Cur concentration increased, the cell viabilities were decreased accordingly in both groups, but no significant difference between Cur–lpNPs and free Cur groups was observed. Furthermore, to investigate the effect of PLGA (poly(lactic-*co*-glycolic acid))–mPEG (methoxyl poly(ethylene-glycol)) and Chol–PEG–RGD copolymer on cell viability, the cytotoxicity of the blank lpNPs was investigated on HEK293 cells. According to Figure 3B, with the increase of copolymer concentration, the viability of HEK293 cells decreased slightly. When the concentration of the copolymer was up to 500 µg/mL, the cell viability was higher than 91%, which suggested that the copolymer showed low cytotoxicity and could serve as a safe drug delivery system.

### 2.3. In Vitro Apoptosis Induction Effect

The *in vitro* apoptosis induction effect of Cur–lpNPs was investigated using the flow cytometric (FCM) assay. FCM assay of Annexin V/PI staining was employed to investigate the apoptosis induction effect of Cur–lpNPs, and both early apoptosis (Annexin V^+^/PI^−^) and late apoptosis (Annexin V^+^/PI^+^) cells were included.

**Figure 4:**
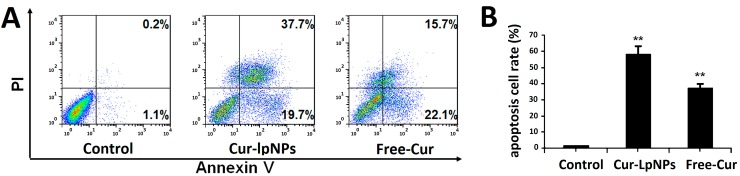
(**A**) Flow cytometric analysis of cells stained with Annexin V-FITC/PI after treatment with Cur–lpNPs or Free Cur; (**B**) Statistic results of apoptosis assays. Data are expressed as means ± SD. from three independent experiments (** *p* < 0.05).

According to Figure 4A,B, the percentage of apoptotic cells in Cur–lpNPs group was 57.4%, which was significantly higher than that in the free Cur (37.8%) and normal saline(NS) (1.3%) groups. No significant difference in early apoptosis between Cur–lpNPs (19.7%) and free Cur (22.1%, *p* < 0.05) group was observed, but late apoptotic cells in Cur–lpNPs group (37.7%) was more than that in the free Cur group (15.7%, *p* < 0.05). The results of the FCM assay suggested that compared with free Cur, Cur–lpNPs induced more apoptotic cells.

### 2.4. Cellular Uptake of Cur–lpNPs by Flow Cytometry Analysis

**Figure 5:**
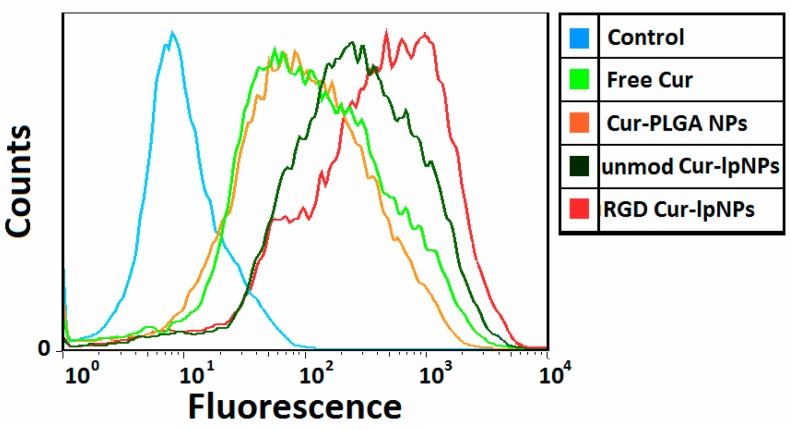
Flow cytometry profiles showed different cellular uptake of blank control (blue), free Cur (green), Cur loaded PLGA NPs (orange), Cur loaded lpNPs with Chol–PEG–RGD (red) and Cur loaded lpNPs without Chol–PEG–RGD (jasper) by human umbilical vein endothelial cells (HUVEC) (containing 40 ng/mL coumarin at 37 °C for 1 h).

A flow cytometry analysis was used to determine the cellular uptake of RGD-modified Cur–lpNPs, unmodified Cur–lpNPs, and Cur–PLGA NPs. Figure 5 showed that the uptake of RGD-modified Cur–lpNPs by human umbilical vein endothelial cells (HUVEC) was more than those of the unmodified Cur–lpNPs and Cur–PLGA NPs after 1 h incubation. The mean fluorescence intensity of the Cur uptaken by HUVECs that were treated with RGD-modified Cur–lpNPs increased by 4.3-fold compared with those which were treated with unmodified Cur–lpNPs and Cur–PLGA NPs. These results confirmed that RGD modification enhanced the uptake of liposomal drug by integrin-over-expressing HUVECs.

### 2.5. In Vivo Anti-Tumor Effect in Subcutaneous Tumor Model

**Figure 6:**
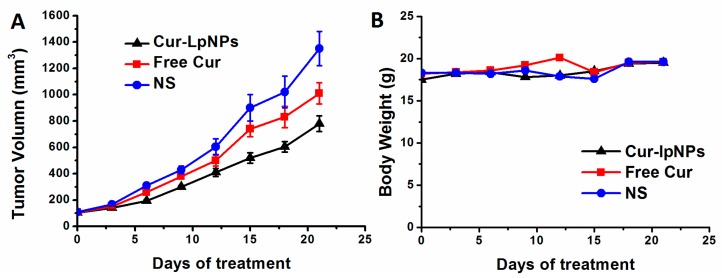
Effects of Cur–lpNPs treatment on primary tumor growth. (**A**) The mean tumor volumes ± SD of 8 mice per every group; (**B**) The mean mice weights ± SD of every group.

The anti-tumor effect of Cur–lpNPs was evaluated in the subcutaneous B16 tumor model. According to Figure 6A, Cur–lpNPs showed greater anti-tumor effects than the free Cur, whereas blank lpNPs showed little antitumor activity. In addition, the treatment of Cur–lpNPs could better increase the life span of tumor-bearing mice than other groups.

To test the anti-tumor effect of Cur–lpNPs in vivo, female BALB/c mice were inoculated subcutaneously with B16 cells. The mice then received intraperitoneally injection (i.p.) of Cur–lpNPs (25 mg/kg) and free Cur (25 mg/kg) or vehicle every three days for 21 days. The results showed that the tumor growth of the Cur–lpNPs treatment group become significantly slowed after 9 days of treatment. Cur–lpNPs substantially inhibited tumor growth, which was significantly lower than that in free Cur, or NS group (Figure 6A). Moreover, Cur–lpNPs treatment was well tolerated and induced no significant weight loss compared with the vehicle group (Figure 6B).

### 2.6. Determination of Apoptosis

**Figure 7:**
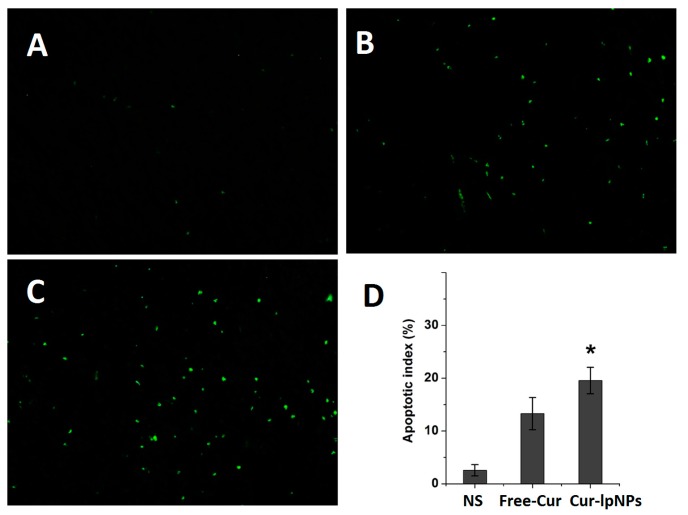
Terminal deoxynucleotidyl transferase-mediated nick-end labeling (TUNEL) immunofluorescent staining of tumors in each group. Representative TUNEL immunofluorescent images (magnification: 40×) of normal saline(NS) (**A**); free Cur (**B**); and Cur–lpNPs (**C**) group; and mean apoptotic index in each group (**D**) (* *p* < 0.05).

Immunofluorescent Terminal deoxynucleotidyl transferase-mediated nick-end labeling (TUNEL) staining assay was used to determine the apoptosis of tumor cells in each group. According to Figure 7A–C, more apoptotic cells were observed in Cur–lpNPs group in comparison with the free Cur or NS groups. As shown in Figure 7D, the apoptotic index in Cur–lpNPs group is 19.55% ± 2.51%, which was higher than that in the free Cur (13.30% ± 3.05%, *p* < 0.001) and NS (2.57% ± 1.06%, *p* < 0.001) groups.

### 2.7. Quantitative Assessment of Microvessel Density (MVD)

**Figure 8:**
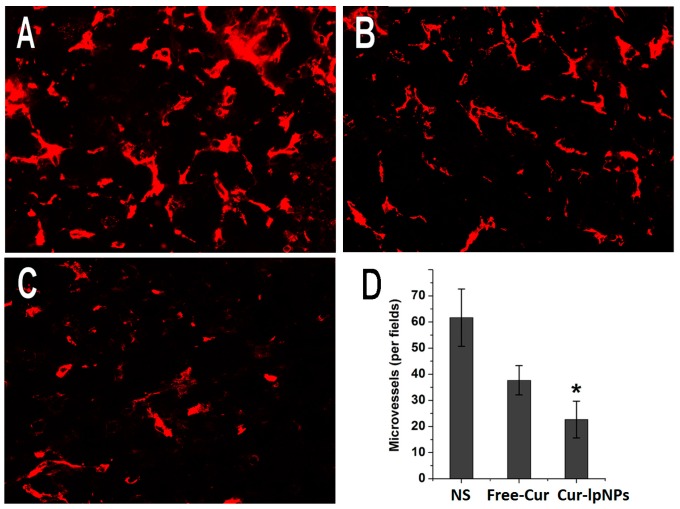
CD31 immunofluorescent staining of tumors in each group. Representative CD31 immunofluorescent images (magnification: 40×) of NS (**A**); free Cur (**B**); and Cur–lpNPs (**C**) group; and mean microvessel density (MVD) in each group (**D**) (* *p* < 0.05).

Immunofluorescent CD31 staining assay was used to quantitatively assess MVD in tumors as a measurement of anti-angiogenesis effects. In Figure 8A–C, fewer microvessels were found in the Cur–lpNPs group than that in other groups. The microvessel density (MVD) was significantly lower in Cur–lpNPs (22.67 ± 7.03) than in free Cur (37.67 ± 5.61, *p* < 0.001) or NS group (61.67 ± 10.95, *p* < 0.001) (Figure 8D).

## 3. Experimental Section

### 3.1. Materials, Cell Lines and Animals

Monomethyl poly(ethylene glycol)–PLGA copolymers (mPEG fragment, *M*_W_ = 2000; PLGA fragment, *M*_W_ = 5000) were acquired from the Changchun Shengbo Ma Biological Material Co., Ltd. (Changchun, China). Methanol (HPLC grade, Fisher Scientific, Loughborough, UK), methyl thiazolyl tetrazolium (MTT, Sigma, St. Louis, MO, USA), propidium iodide (PI, Sigma, St. Louis, MO, USA), 1-phenyl-2-thiourea (PTU, Sigma, St. Louis, MO, USA), and tricaine (Sigma, St. Louis, MO, USA) were used without further purification. All the materials used in this article were of analytic reagent (AR) grade and were used as received.

HUVEC cells and B16 cells were purchased from the American Type Culture Collection (ATCC; Rockville, MD, USA). The HUVEC cells grew in Roswell Park Memorial Institute 1640 medium (RPMI 1640, Gibco, Waltham, MA, USA) supplemented with 10% fetal bovine serum (FBS, Gibco, Waltham, MA, USA), whereas the B16 cells grew in Dulbecco’s modified Eagle’s medium (DMEM, Gibco, Waltham, MA, USA) supplemented with 10% FBS. All above cells were maintained at 37 °C in a humidified incubator containing 5% CO_2_.

BALB/c mice (6 weeks) were used for the *in vivo* anti-tumor experiments. The animals were purchased from the Laboratory Animal Center of Sichuan University and were housed at controlled temperatures of 20 to 22 °C, relative humidity of 50% to 60%, and 12 h light-dark cycles. The animals were provided with standard laboratory chow and tap water *ad libitum*. All animal procedures were performed following the protocol approved by the Institutional Animal Care and Treatment Committee of Sichuan University (project identification code: WCHSIRB-D-2014-021, 28 February 2014).

### 3.2. Preparation and Characterization of Cur–lpNPs

The Cur–lpNPs were prepared by the W/O/W double emulsification method, according to the standard procedure we reported previously [14]. Unless otherwise mentioned, all the experiments were conducted by varying one of the parameters while keeping all the other process parameters at a set of standard conditions, as follows. First, 40 mg mPEG–PLGA copolymer, 10 mg lipids mixture (lecithin/cholesterol = 5/1, *w*/*w*), and 500 µg Chol–PEG–RGD were dissolved in 1 mL dichloromethane as the organic phase. Then, the organic phase was emulsified with the internal aqueous phase containing 0.2 mL Cur ethanol solution (10 mg/mL) using the JY92-IIDN microtip probe sonicator (Scientz Co., Ltd, Ningbo, Jiangsu, China) in ice bath at 55 W for 1 min with 5 s pulse-on and 5 s pulse-off. After the formation of W/O coarse emulsion, 4 mL of 1% PVA (*M*_W_ = 30–70 KD) solution was added into the coarse emulsion to further get W/O/W double emulsion via sonication (65 W for 1 min with 5 s pulse-on and 5 s pulse-off). Then, the organic solvent was immediately removed through rotary vacuum evaporation. The solution was then centrifuged for 70 min (1.20 × 10^4^ g/min) at 4 °C to remove the PVA and free Cur. The precipitate was dissolved in PBS (pH 7.4, 0.01 M). The obtained Cur–lpNPs were stored at 4 °C for further evaluation.

When the Cur payload increased to 7%, a slight increase in DL% and a sudden decrease in its EE% (70.7%) were observed. This result suggested that when the drug/polymer ratio was higher than 5% (*w*/*w*), the drug cannot be fully entrapped in the nanoparticles, hence, the optimal drug/polymer ratio was 5%, with good drug loading capacity (4.80%) and high encapsulation efficiency (96%). The particle size distribution and zeta potential of Cur–lpNPs were determined using Malvern Nano-ZS 90 laser particle size analyzer (Malvern, Worcestershire, UK). The results were the mean of the three different samples, and all the data were expressed as the mean ± standard deviation (SD). The morphological characteristic of Cur–lpNPs was examined using transmission electron microscope (TEM, H-6009IV, Hitachi, Japan). The sample of Cur–lpNPs was negatively stained with phosphotungstic acid before the test.

The DL and EE of Cur–lpNPs were determined via high performance liquid chromatography (HPLC, Waters Alliance 2695, Milford, MA, USA) instrument. The detection was taken on a Waters 2996 detector, and the chromatographic separations were performed on a reversed phase C_18_ column (4.6 × 150 mm to 5 µm, Inertsil/WondaSil, Torrance, CA, USA). The DL and EE of Cur–lpNPs were calculated according to the following Equations (1) and (2):

DL = Drug/(Drug + Polymer) × 100%
(1)

EE = Drug in NPs/drug in feed × 100%
(2)

### 3.3. In Vitro Cytotoxicity

To investigate the cytotoxicity of Cur–lpNPs and free Cur, MTT assays were preformed on B16 cells. B16 cells cultured in 96-well plates were treated with a series of Cur–lpNPs or free Cur for 48 h. The mean percentage of cell survival relative to that of control cells was determined from the data of six individual experiments, and all the data were expressed as mean ± SD.

### 3.4. Cellular Apoptosis Assay

The apoptosis induction assay of Cur–lpNPs and free Cur were studied on B16 cells. B16 cells were plated in 6-well plates and grown for 24 h. The cells were exposed to media containing 20 ng/mL of Cur–lpNPs, free Cur, or blank solvents for 48 h. Then, the cells were fixed with pre-chilled 70% ethanol for 30 min and stained with 0.5 mL of PI (5 µg/mL in PBS) for 10 min. Apoptotic cells, which demonstrated cytoplasmic and nuclear shrinkage and chromatin condensation, were observed under fluorescence microscopy (TE2000-U, Nikon, Tokyo, Japan).

Flow cytometric (FCM) assay was used to confirm the apoptotic induction effect of Cur–lpNPs. The apoptosis of B16 cells treated with Cur–lpNPs, free Cur, or blank solvents were determined using FITC-conjugated Annexin V/PI (BD PharMingen, Franklin Lakes, NJ, USA) staining by FCM (BD FACSCalibur, Franklin Lakes, NJ, USA), both in the early apoptotic (Annexin V^+^/PI^−^) and late apoptotic (Annexin V^+^/PI^+^) groups.

### 3.5. Cellular Uptake of Cur–lpNPS by Flow Cytometry Analysis

An aliquot of 1.5 mL HUVEC cells suspension (6 × 10^4^ cells/well) was seeded in a six-well tissue culture plate (Corning Incorporated, Corning, NY, USA) and was incubated for 24 h at 37 °C. Then, 30 µL RGD-modified Cur–lpNPs, unmodified Cur–lpNPs, and Cur–PLGA NPs were added into each well, and the final concentration of Cur was 40 ng/mL. The plates were incubated at 37 °C for another 1 h, after which the medium was discarded, and the cell monolayer was suspended by the treatment with trypsin and washed three times with cold PBS. Then, the cell samples were examined using a flow cytometer (EPICS Elite ESP, Beckman Coulter, Brea, CA, USA). The intracellular Cur was excited with an argon laser (488 nm), and the fluorescence was detected at 525 nm. The files were collected of 10,000 gated events.

### 3.6. Subcutaneous Tumor Mouse Model

To investigate the anti-tumor activity of Cur–lpNPs, a subcutaneous B16 tumor mouse model was used. About 100 µL of B16 cell suspension (5 × 10^5^ cells) were injected subcutaneously in the right flank of mice at day 0, and when the tumors were palpable at day 4, the mice were randomized into four groups (12 mice per group). At days 4, 9, and 14, the mice were injected intravenously with 100 µL of NS (control), free Cur (2 mg/kg), or Cur–lpNPs (2 mg/kg), respectively. Each mouse in each group was used for the tumor growth inhibition assay, and the tumor size was measured every three days using calipers. The tumor volume was calculated using the Equation (3), where *vol* is the tumor volume, *a* is the length of the major axis, and *b* is the length of the minor axis. The mice in the control group began to die at day 29, and the other mice were sacrificed via cervical vertebra dislocation.

*vol* = 0.52 × (*a* × *b*^2^) (3)

### 3.7. Detection of Apoptosis

The tumor tissue specimens were harvested, fixed in paraformaldehyde (4 wt. %), embedded in paraffin, and sectioned. Terminal deoxynucleotidyl transferase-mediated nick-end labeling (TUNEL) staining was preformed using an *in situ* cell death detection kit (Roche, Basel, Switzerland) according to the manufacturer’s protocol. Five equal-sized fields of sections were randomly chosen and analyzed, and the apoptotic index was calculated as a ratio of the apoptotic cell number to the total tumor cell number in each high-power field.

### 3.8. Quantitative Assessment of MVD

Immunofluorescent analysis of neovascularization in tumor tissue was used to determine the anti-angiogenesis activity of Cur–lpNPs. The frozen sections of tumors were fixed in acetone, stained with rat anti-mouse CD31 polyclonal antibody (1:50; BD Pharmingen™, Franklin Lakes, NJ, USA), and incubated with a FITC-conjugated second antibody (Abcam, Cambridge, MA, USA). The MVD was determined by counting the number of microvessels per high-power field in the sections using a fluorescence microscopy (TE2000-U, Nikon, Tokyo, Japan).

### 3.9. Statistical Analysis

The statistical analysis was performed using the SPSS 15.0 software (Chicago, IL, USA). Comparisons of tumor volume, tumor weight, and number of tumor nodules were performed using one-way analysis of variance (ANOVA). Survival curves were generated based on the Kaplan–Meier method, and the statistical significance was determined using Mann–Whitney *U*-tests. A *p* value <0.05 on a 2-tailed test was considered statistically significant.

## 4. Conclusions

The RGD functional Cur–lpNPs, with small particle size and high encapsulating efficiency, were prepared. The RGD functional Cur–lpNPs have been successfully developed as a potential drug delivery system, and the poorly soluble anti-tumor drug Cur as model is loaded into the nanoparticles. The nanoparticles are spheroids with regular shape, a size within 200 nm, and showed increased intergrin receptor positive cellular uptake and sustained *in vitro* drug release behavior. The *in vitro* cytotoxicity was evaluated on the B16 cell lines via the MTT assay and demonstrated that Cur–lpNPs significantly enhanced the cytotoxicity of Cur for melanoma cells and increased apoptosis induction. Furthermore, Cur–lpNPs could suppress tumor growth and prolong survival in a subcutaneous B16 tumor model. Although further investigation on this lpNP drug delivery system is required, the findings of our study represent an extension for the use of lipid-shell and polymer core nanoparticles as a potential strategy for novel drug delivery systems.
